# Separating Teeth

**Published:** 1873-09

**Authors:** 


### EDITORIAL, ETC.

---

*Separating Teeth.*—At the late meeting of the "Illinois State Dental Society," the following discussion arose concerning "Arthur's method:"

Dr. Crouse, of Chicago, remarked that he should advise members to get Arthur's book, and study it, although there was a little appearance of quackery and "gilt edge" about the work, yet, being partially addressed to the public, it might save us a great deal of trouble if our patients would read it.

Separating teeth is a matter of great importance. The first thing to determine is how to effect the separation. In order to be of benefit, it must be permanent. It is not necessary always to make these separations V-shaped, and sometimes, with bicuspids, as in certain cases of irregularity, it is better to have the base of the triangle on the outside, because the pick can be best used if teeth are left in that manner. In the lower teeth, particularly, he should generally have the widest space on the outside, as the saliva from the ducts under the tongue is in great abundance on the inside, and, hence, can wash out such spaces better; aside from this, it is almost impossible to remove food from between the lower teeth with a pick, if such spaces are made largest from the inside. Arthur does not describe his process minutely enough.

In irregular teeth, the speaker should, in the majority of cases, leave a shoulder, and not make a V-shaped separation.

In front upper teeth, separations may often be made from the palatal side, with cone-shaped burrs, so as to leave the front edges in contact, and thus retain the natural appearance of the teeth.

The proper time for these operations is just as soon as decay commences; parents must be taught that these operations are essential to prevent the necessity of filling within a short time. In the molars and bicuspids there is an objection to these operations, on account of the food pressing in, and being inconvenient in mastication.

The prejudice against these operations must first be removed from the profession, as we are responsible for its existence among the people.

Dr. Miner, Davenport, Iowa—It used to be advocated long years ago that superficial decay should be filed away. In the mouth of a prominent gentleman in the profession, teeth that had been treated in this manner thirty years ago, have not since decayed.

Dr. Chase, St. Louis, was pleased with the amount of vital action acknowledged in the paper. Arthur's plan is, not only to operate upon teeth which already are decayed, but also on those which are perfectly sound, to prevent its occurrence. Eight years ago a lady now living in St. Louis, then a patient of Dr. Arthur's, had her teeth treated by him for prevention. They were at the time perfectly sound; teeth immediately became sensitive, operation was repeated two or three times afterwards, and teeth are to this day tender. The lady has a sister two years younger, whose teeth are perfectly sound; Dr. A. desired to operate upon them in same manner, but the lady being frightened by her sister's experience, declined. Now, to-day, these teeth that have not been operated upon at all, are perfectly sound.

This is a case of positive injury. Merely states this case to put people on their guard. Where decay has already commenced, the operation is allowable. Is willing to be put on record as against the theory as advocated in that book.

The central incisors on the palatal side can be cut away with good advantage, and without the danger, as in bicuspids and molars, but this has been generally practised a long while. Dr. Arthur's method requires constant attention after the operations ; if just as much care is given the teeth under other circumstances, decay would be just as much prevented.

Where there is great pressure of the teeth, the speaker advocates extraction of the second bicuspid, as in children thirteen or fourteen years old. Has practiced this, and knows the first molar will move bodily forward, and not tilt, and also the first bicuspid will move backward. It will also increase the room for the wisdom teeth.

Dr. Marsh, Chicago—Is not converted to Dr. Arthur's method ; recommends the use of floss silk to keep teeth clean. In crowded arches expands by spreading bicuspids; fills carious cavities with gold.

Dr. Kulp, Davenport, Ia.—Is now treating a patient who had his teeth filed apart for the purpose of preventing decay. Three or four years ago he had all the proximal surfaces filled with amalgam in the East, which amalgam fillings have since been replaced with gold.

On one side a tooth had been lost, and no filling had been done, there was no decay on that side. He concludes that the cutting away is not safe practice in all cases. It is unsafe in weak constitutions, and between bicuspids and molars.

Dr. Harlan, Chicago.—Teeth should be separated at an early age, and the separations polished frequently afterwards, for at least three years; thoroughness in polishing is necessary ; a coarse file or corundum is not sufficient, and square shoulders must not be left, as it is not possible to keep such surfaces clean. The discerning dentist must determine where, and where not, to operate in this manner.

Dr. Low, Chicago.—The more he reads Arthur's work the less he agrees with him. Changing the shape of teeth injures the design of mastication. Cleanliness is essential, without cutting away the material of the teeth.

Dr. Black, Jacksonville, Ill.—Was at first almost disgusted with the book, but after studying closely the controlling idea contained in it (prevention of decay on proximal surfaces) has modified his view. We may do harm in some improper cases, but a great deal of good in proper ones. Decay occurs at points where there is a statical condition of the fluids; if a separation be effected at these points so as to secure free and continuous motion of the fluids, it will be beneficial. In changing the shape, square shoulders must not be left, especially at the gingival border, for the gum to lap over and become irritated, and give out acid secretions. There is an age when all decay ceases; never had decay commence in his own mouth after he was seventeen years old. The fluids of the mouth are in such condition that the teeth could not decay; vital action or no vital action, the phosphate of lime does not dissolve.

